# PEDOT:PSS-based Multilayer Bacterial-Composite Films for Bioelectronics

**DOI:** 10.1038/s41598-018-33521-9

**Published:** 2018-10-16

**Authors:** Tom J. Zajdel, Moshe Baruch, Gábor Méhes, Eleni Stavrinidou, Magnus Berggren, Michel M. Maharbiz, Daniel T. Simon, Caroline M. Ajo-Franklin

**Affiliations:** 1 Department of Electrical Engineering and Computer Sciences, University of California, Berkeley, Berkeley, California United States of America; 2 Molecular Foundry, Lawrence Berkeley National Laboratory, Berkeley, California United States of America; 3 Laboratory of Organic Electronics, Linköping University, Norrköping, Sweden; 4 Department of Bioengineering, University of California, Berkeley, Berkeley, California United States of America; 5 Chan Zuckerberg Biohub, San Francisco, California United States of America; 6 Molecular Biophysics and Integrated Bioimaging Division, Lawrence Berkeley National Laboratory, Berkeley, California United States of America

## Abstract

Microbial electrochemical systems provide an environmentally-friendly means of energy conversion between chemical and electrical forms, with applications in wastewater treatment, bioelectronics, and biosensing. However, a major challenge to further development, miniaturization, and deployment of bioelectronics and biosensors is the limited thickness of biofilms, necessitating large anodes to achieve sufficient signal-to-noise ratios. Here we demonstrate a method for embedding an electroactive bacterium, *Shewanella oneidensis* MR-1, inside a conductive three-dimensional poly(3,4-ethylenedioxythiophene):poly(styrenesulfonate) (PEDOT:PSS) matrix electropolymerized on a carbon felt substrate, which we call a multilayer conductive bacterial-composite film (MCBF). By mixing the bacteria with the PEDOT:PSS precursor in a flow-through method, we maintain over 90% viability of *S. oneidensis* during encapsulation. Microscopic analysis of the MCBFs reveal a tightly interleaved structure of bacteria and conductive PEDOT:PSS up to 80 µm thick. Electrochemical experiments indicate *S. oneidensis* in MCBFs can perform both direct and riboflavin-mediated electron transfer to PEDOT:PSS. When used in bioelectrochemical reactors, the MCBFs produce 20 times more steady-state current than native biofilms grown on unmodified carbon felt. This versatile approach to control the thickness of bacterial composite films and increase their current output has immediate applications in microbial electrochemical systems, including field-deployable environmental sensing and direct integration of microorganisms into miniaturized organic electronics.

## Introduction

Electrochemical biosensors couple selective biological processes to electronic readouts and are of interest for environmental sensing applications due to their rapid response times, relatively low power consumption, and the variety and specificity of biomolecular sensing afforded by biological receptors^1,2^. Electroactive bacteria in microbial electrochemical systems (MESs) are especially attractive for use in biosensing since they directly couple specific biological processes to a readily measured current or potential readout^3,4^. Recent examples use *Shewanella oneidensis* to produce an electric current in response to arsenic, arabinose, or organic acids^2,5,6^. However, electroactive bacteria commonly used for biosensing, including *S. oneidensis* and engineered *Escherichia coli*, do not form thick biofilms and produce relatively low currents and therefore feature a small signal-to-noise ratio (SNR) for the small anode volumes desired for environmental deployment^5–8^. Because the anode interfaces directly with bacteria, it is of great interest to develop a modification scheme that increases the density of cell-anode attachment beyond that of natural biofilms and also ensures that all cells are able to transfer electric charge to the anode effectively. To ensure deployability, any modification should not significantly increase the anode’s volume or reduce analyte permeability.

Because the effective surface area of the anode limits the number of bacteria that make electrical contact, many modification approaches aim to maximize the surface area to volume ratio of anodes. These methods include the use of high effective area porous carbon structures such as carbon felt (CF), carbon cloth, carbon nanotubes, graphene foam, or precious metals such as gold nanoparticles and graphene-gold composites^9–13^. However, the inherent hydrophobicity of carbon-based structures is incompatible with robust microbial adhesion, and these methods are also restricted to use with species that are capable of producing a robust biofilm^14^. Polymer-based materials, such as polyaniline or polypyrrole (PPy), have also been used in an attempt to increase power density in MESs^15,16^. In addition, encapsulation of *S. oneidensis* by PPy has been recently demonstrated to enhance the electron transfer rate from bacteria to anodes while maintaining bacterial viability^17^. However, these materials do not significantly increase the density of the thin biofilm naturally formed by *S. oneidensis*^18^. Thus, existing methods do not address the need to increase the density of current-producing microorganisms in devices.

The highly conducting and biocompatible organic polymer, poly(3,4-ethylenedioxythiophene) (PEDOT), has seen broad adoption in biological-electronics interfaces^19–23^. PEDOT has become a *de facto* standard material for organic bioelectronics due to its well-defined redox properties, large volumetric capacitance, mixed electronic/ionic conduction, and stability in water when mixed with poly(styrenesulfonate) (PSS) as a dopant. Chemically-polymerized and electrospun PEDOT has been used to increase the surface area and conductivity of anodes in MESs and microbial fuel cells (MFCs)^24–26^. Thin films (~500–900 nm) of gram-negative bacteria on indium-tin-oxide (ITO) have also been produced using PEDOT, but only the outermost layers contained live electroactive bacteria, limiting the current production^27^. This work suggests that keeping electroactive bacteria viable throughout a thick multilayer conductive biofilm is key towards increasing the bacterial density and current production at the anode beyond that of present methods.

In this work, we have developed a multilayer conductive bacterial-composite film (MCBF) produced by embedding of living *S. oneidensis* in electropolymerized nutrient-permeable PEDOT:PSS and its simultaneous immobilization on a porous CF substrate. The resulting MCBFs show a 20-fold increase in steady state current production over unmodified CF anodes when used in standard MESs. The scalable anode fabrication process, improved electron transfer through a 3D conductive biomatrix, high viability, and the ability to use strains that do not form thick native biofilms demonstrate an important advance towards establishing advanced, field-deployable anode modifications for MESs, or direct integration of microorganisms into miniaturized organic electronic devices.

## Results

### A scalable process encapsulates bacteria while preserving viability

**Figure 1:**
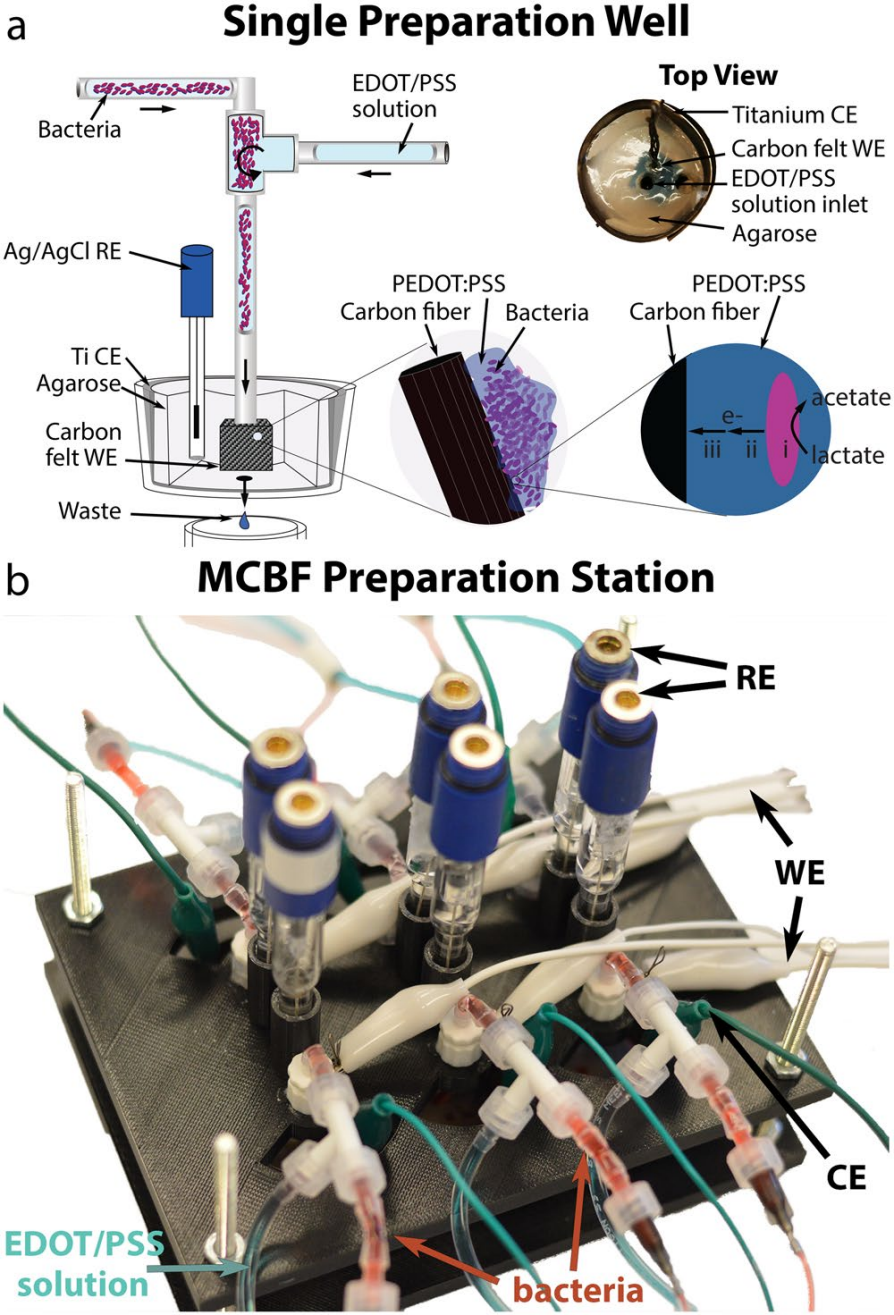
Electrode preparation set-up for viable multilayer conductive bacterial-composite film production. (**a**) Schematic of the electropolymerization system, including a photograph of a single well. The electron flow in the final structure is (i) reduction of lactate to acetate by bacteria, (ii) transfer of electrons from bacteria to the PEDOT:PSS scaffold, and (iii) conduction of electrons through PEDOT:PSS scaffold to CF substrate. (**b**) Isometric view of the complete MCBF preparation station for parallel electropolymerization of six bio-anodes.

To improve the volumetric current density produced by whole cell sensors, we sought to embed *S. oneidensis* into a three-dimensional matrix of PEDOT:PSS around carbon felt (CF) (Fig. 1a). This method needed to meet several key requirements: i) the vast majority of the bacteria must remain viable, ii) each bacterial cell should be connected by conductive material to the CF surface, iii) the matrix must permit rapid ion mobility and small molecule diffusion, and iv) it should permit parallel and reproducible fabrication.

Initial experiments showed that exposure of *S. oneidensis* to 10 mM 3,4-ethylenedioxythiophene (EDOT) for 16 hours at 4 °C reduced the viability to below 50% (Supplementary Figure S1). Therefore, we developed a new electropolymerization protocol that minimized bacterial exposure to EDOT monomer. We used separate reservoirs for the EDOT/PSS precursor solution and bacterial suspension, pumped them so that they mixed in a T-junction just before their introduction to the preparation well, and allowed excess non-polymerized solution to exit this well through a small drainage hole (Fig. 1a). Additionally, we carried out the electropolymerization at 4 °C to keep the bacteria in a dormant state. These precautions maximized bacterial viability throughout the extended electropolymerization process (data shown below).

To ensure PEDOT:PSS was electropolymerized around each cell sufficiently while simultaneously confining the bacteria to the CF surface, we introduced a concentrated mixture of EDOT/PSS solution and bacteria directly to the anode. More specifically, the electropolymerization solution was introduced at a volumetric flow rate 15 times higher than that of the bacterial solution. As a result, a large volumetric charge of 1562 mC cm^−3^ was delivered during the electropolymerization process (Supplementary Figure S2), corresponding to the oxidation of EDOT and the subsequent formation to PEDOT, as well as to the charge doping of PEDOT^28^. The flow-through waste accumulated a substance that appeared dark blue, the hallmark color of PEDOT, providing further evidence of PEDOT polymerization^29^. A permeable agarose gel surrounding the CF slowed diffusion, confining both bacteria and the electropolymerization solution to the CF during electropolymerization (Fig. 1a, Top View).

Lastly, to make the modification process more scalable, we built an MCBF preparation station (Fig. 1b, Supplementary Figure S3) capable of simultaneously producing six MCBFs with equal flow-and deposition rates. As a control, bacteria can be omitted and PEDOT:PSS electropolymerized around the CF, resulting in an abiotic multilayer conductive film (MCF). Our setup is a step towards a platform that scalably fabricates multiple bio-anodes for MESs. The end result of the electropolymerization process was a set of functional electrodes consisting of *S. oneidensis* embedded in a PEDOT:PSS-covered CF bulk, the whole electrode being encircled by a 2 mm-thick layer of agarose gel for mechanical support.

### Electropolymerization yields viable bacteria encapsulated in MCBFs

To probe the viability of the bacterial cells embedded in the MCBFs, we sliced the MCBF with a scalpel immediately after the electropolymerization process and performed a live/dead viability stain (Supplementary Figure S4). The bacterial viability at this stage was 91% ± 5% across three biological replicates. Thus, the PEDOT:PSS electropolymerization method preserves high bacterial viability, meeting the first key requirement for the MCBF structure. We suggest that limiting the exposure of *S. oneidensis* to EDOT monomer increased overall viability from 50% to >90%.

### Electropolymerization increases the electrochemically active volume of the electrode

**Figure 2:**
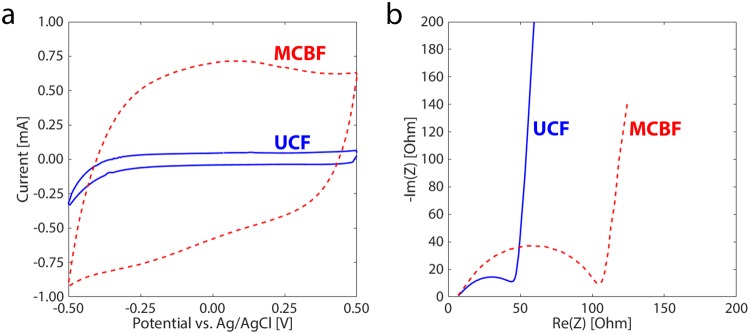
Electropolymerization greatly increases the specific capacitance in MCBFs relative to UCFs. (**a**) Cyclic voltammograms and (**b**) Nyquist plots measured for unmodified CF before (solid blue lines), and for MCBF after the electropolymerization process (red dashed lines).

Our electropolymerization process was designed to generate a large electrochemically active volume on the bio-electrode. To estimate the electrochemically active surface area, we used cyclic voltammetry (CV) to measure the capacitive current of untreated CF anodes (UCFs), abiotic multilayer conductive films (MCFs), and MCBFs, each with the same approximate volume of 0.635 cm^3^. The UCF had a capacitive current (Fig. 2a) corresponding to a calculated volumetric capacitance of 1.75 mF cm^−3^ (details of the calculation are in the Supplementary Information), indicative of surface-only electroactivity. In contrast, both the abiotic MCF and MCBF samples featured significantly higher CV currents (Fig. 2a, Supplementary Figure S5, respectively) after electropolymerization, corresponding to representative volumetric capacitances of 19.21 and 20.37 mF cm^−3^, respectively. This 11-fold increase in volumetric capacitance is consistent with the formation of multiple PEDOT:PSS layers on the UCF surfaces and in the voids between individual fibers. Indeed, high capacitive currents are typical for thick PEDOT:PSS-based electrodes due to the mixed electronic-ionic conduction in the bulk of PEDOT:PSS films^30,31^. Moreover, the almost negligible difference between the volumetric capacitance values of the MCF and the MCBF indicate that the presence of bacteria did not influence the electrochemically active surface area.

An ideal anode would have a very low charge transfer resistance (*R*_*CT*_) to ensure good electron transfer. To determine *R*_*CT*_ values, we performed electrochemical impedance spectroscopy (EIS) on the same samples analyzed by CV. We note that the trends in EIS data before and after electropolymerization did not differ between MCBFs and MCFs (Supplementary Figure S5) therefore, the effect of bacteria on these data is not considered here. The Nyquist plot of a UCF shows the typical semicircle and tail indicative of both capacitive and resistive contributions to the impedance (Fig. 2b). In the case of a representative MCBF, we can see a somewhat larger semicircle, representing a 2.40x increase in *R*_*CT*_, from 43 Ω to 103 Ω, where the values were derived from fitting a simple electrical circuit model to the Bode plots (Supplementary Figures S6, S7; details of the equivalent circuit modeling are given in the Supplementary Information). This small, 60 Ω increase in *R*_*CT*_ may indicate a series resistance caused by the incorporation of PEDOT:PSS clusters. In any case, this minor increase in *R*_*CT*_ would not affect measured biocurrents with MCBFs for current levels practically achievable in small-volume MESs. Thus, we conclude that the volumetric capacitance of MCBFs increased dramatically relative to UCFs while only showing a negligible increase in electrical resistance. Together these properties should ensure a higher biocurrent in MCBF-based MESs compared to those achievable in UCF-MESs.

### Electropolymerization embeds a high density of bacteria

**Figure 3:**
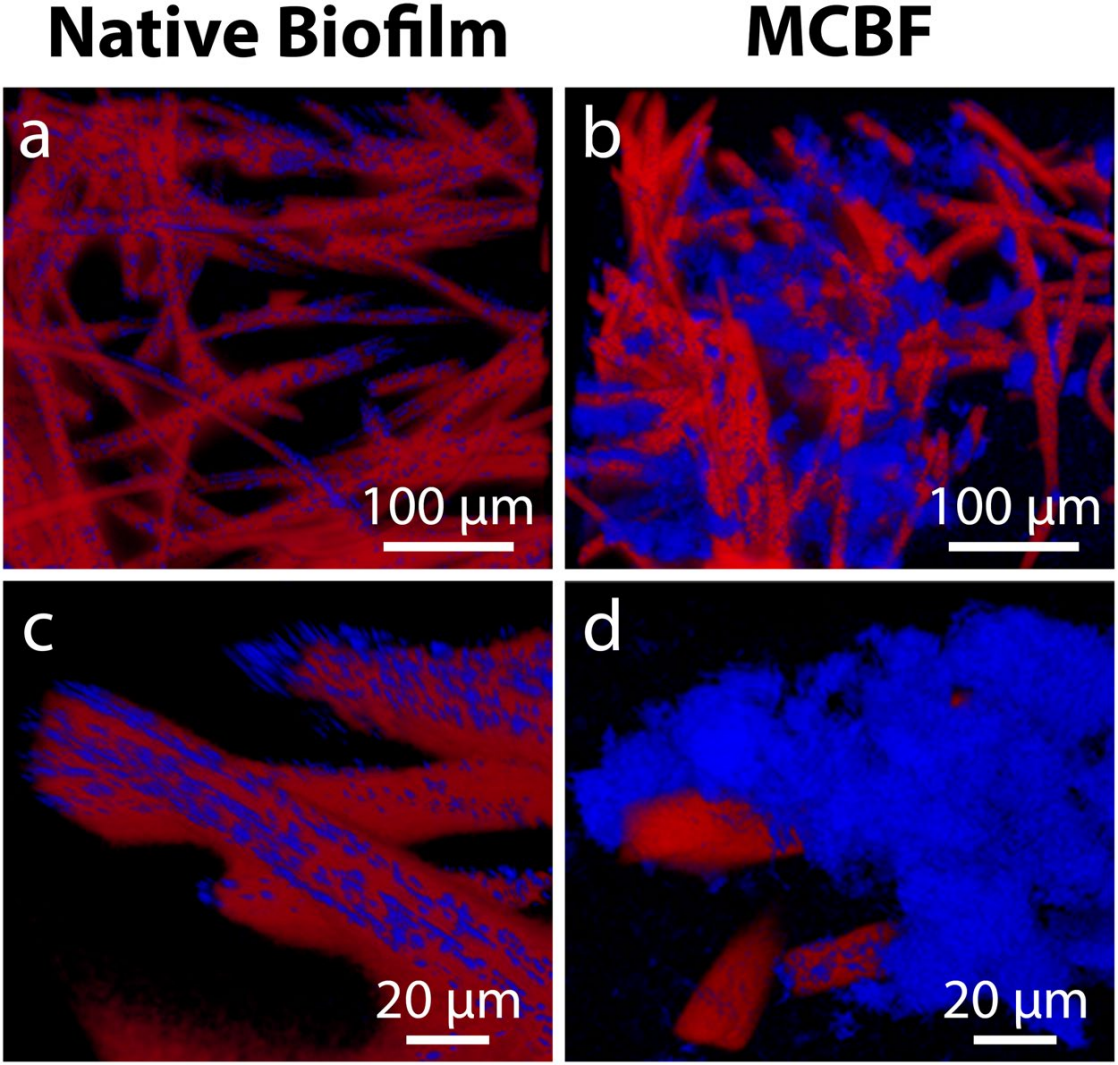
Multilayer conductive bacterial-composite films (MCBFs) are thicker than native *S. oneidensis* MR-1 biofilms on unmodified CF (UCF). Confocal microscopy images of cross sections of (**a** and **c**) native biofilm on UCF and (**b** and **d**) MCBF. Red color indicates CF and blue *S. oneidensis*.

To understand how bacteria are embedded inside of the PEDOT:PSS bulk, we examined slices of a UCF with a native biofilm and an MCBF via a confocal microscope. These images reveal that bacteria covered the UCF fibers with low densities that varied locally and frequently settled into the deep transverse grooves along the length of each fiber (Fig. 3a,c, bacteria in blue). In addition, between the UCF fibers were large voids which were not filled by bacteria. This limited coverage of the volume by bacteria within the UCF electrode was a result of surface-only monolayer attachment. In contrast, many more bacteria were present in the MCBF, and they were concentrated in large clusters (Fig. 3b,d). Moreover, the encapsulated bacterial clusters were both on the CF fibers and in the voids between the CF fibers to form high density multilayer bacterial structures up to 80 µm thick. Thus, the process described above demonstrates the enclosure of bacteria into a three-dimensional matrix significantly thicker than its native biofilm.

### Multilayer bacterial structure is revealed by scanning electron microscopy

**Figure 4:**
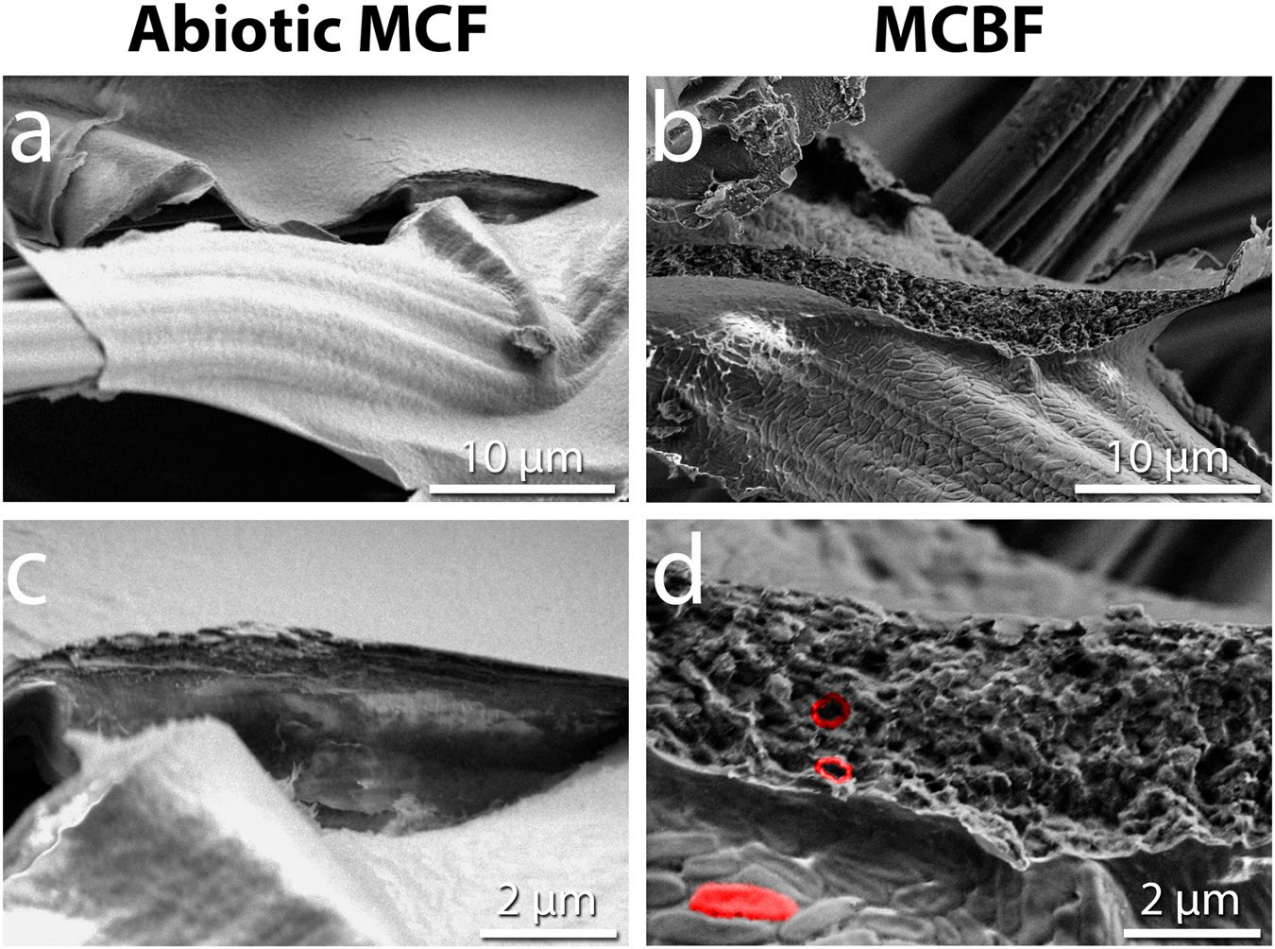
Electropolymerization of living *S. oneidensis* MR-1 embeds the bacteria inside a multilayer conductive bacterial-composite film. Scanning electron micrographs of a section of (**a** and **c**) an abiotic multilayer conductive film (MCF) showing smooth internal surfaces of PEDOT:PSS on CF, and (**b** and **d**) of an MCBF showing high density of bacteria within PEDOT:PSS layers. (**d**) One bacterium on the external surface of the MCBF is shaded in red, while two bacteria-sized voids internal to the MCBF are outlined in red.

To probe the morphology within the multilayer structures on the sub-micron level, we desiccated and sectioned three abiotic MCF and three MCBF electrodes and examined them by scanning electron microscopy (SEM). Normally, fibers of UCF are held together in well-separated thick bundles (Supplementary Figure S8). In contrast, a close look at an abiotic MCF section reveals smooth solid structures with uniform cross sections stretching between fibers of CF (Fig. 4a,c). Since the layers did not charge quickly during electron microscopy, we interpret these conductive structures as layers of PEDOT:PSS. On the other hand, SEM of the MCBF reveals a porous and rough internal surface replete with rod-shaped cavities that are roughly 1–2 µm in dimension (Fig. 4b,d). We interpret these cavities as the imprints of embedded bacteria after they dehydrated and shrank during the SEM sample preparation process. The flat dehydrated *S. oneidensis* structures are easily seen on the top surface of the MCBF films and comparable in size to the cavities within the section (Fig. 4b). Thus, we conclude that bacteria in the MCBF are encapsulated by PEDOT:PSS layers, creating closely positioned, highly integrated, multilayer bacterial-composite films. Consequently, our MCBF preparation process does not only encapsulate high densities of bacteria into a three-dimensional matrix, but also embeds them into a conductive multilayer structure.

### *S. oneidensis* transfers electrons to PEDOT:PSS via direct and riboflavin-mediated mechanisms

**Figure 5:**
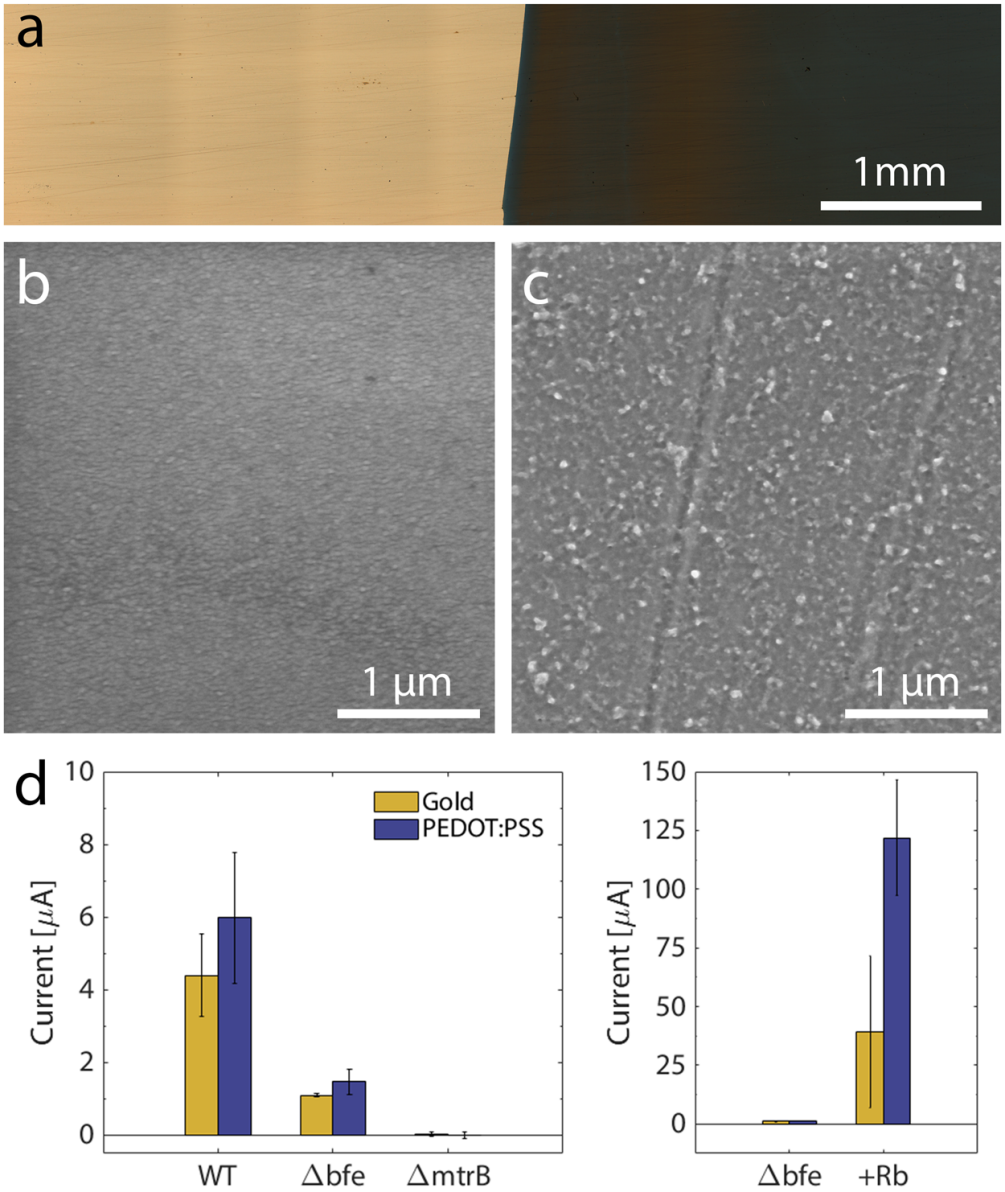
*S. oneidensis* MR-1 transfers metabolic current through PEDOT:PSS by both direct and flavin-mediated electron transfer. (**a**) 5x magnification of Au/PEDOT:PSS deposited on slide. (**b**) SEM of gold thin film surface. (**c**) SEM of PEDOT:PSS film. (**d**, left) Average current after 12 hours for WT, Δ*mtrB* and Δ*bfe* strains on gold and PEDOT:PSS films and (**d**, right) the Δbfe current production before and 12 hours after addition of 10 μM riboflavin (Rb).

The large majority of *S. oneidensis* MR-1 cells are embedded within the PEDOT:PSS matrix without any direct contact with the CF (Figs 3, 4). To establish whether these distant cells could transfer electrons through PEDOT:PSS to CF, we first fabricated a PEDOT:PSS thin film on a Au electrode and then monitored current produced from *S. oneidensis* attached to this film. Electropolymerization on Au (see Experimental Section) consumed 0.206 mC cm^−2^ of charge (Supplementary Figure S9) and yielded a thin, characteristically dark-blue PEDOT:PSS film (Fig. 5a)^29^. Surface profilometry determined that the thickness of the PEDOT:PSS layers varied between 120 to 220 nm, and SEM images revealed a bulbous structure of the PEDOT:PSS films that completely covered the smooth Au (Fig. 5b,c). Thus the geometry of the PEDOT:PSS/Au electrode requires that any current generated by the bacteria is transferred through the PEDOT:PSS to be measured.

*S. oneidensis* MR-1 transfers electrons to CF electrodes either by direct electron transfer (DET, ~20–30% of the total current) or via a riboflavin-mediated mechanism (~70–80% of the total current)^32,33^. To probe whether *S. oneidensis* can transfer electrons through PEDOT:PSS with either mechanism, we compared the current collected by Au and PEDOT:PSS/Au electrodes in bioreactors lacking bacteria or containing three different strains: wild type (WT) *S. oneidensis* (which uses both mechanisms), a riboflavin-deficient mutant Δ*bfe* that only performs DET, and the current-deficient mutant Δ*mtrB*. The baseline current just before addition of bacteria was subtracted from the steady-state current produced 12 hours after the injection of bacteria for comparison (Supplementary Figure S10). As expected, the Δ*mtrB* strains did not produce appreciable current above the background, nor did abiotic reactors with injected riboflavin, which rules out abiotic current production by PEDOT:PSS or riboflavin (Fig. 5d). Wild type cultures transferred significant current, ~5 µA, to both electrodes. Interestingly, the PEDOT:PSS/Au electrode harvested significant current from the Δ*bfe* mutant (Fig. 5d), although it was ~4-fold lower than the WT strain. This observation indicates that *S. oneidensis* can directly transfer electrons to PEDOT:PSS. Additionally, the consistent current ratios between the WT and Δ*bfe* strains on both materials suggests that DET contributes roughly the same electron transfer flux from *S. oneidensis* MR-1 to planar PEDOT:PSS, Au, and CF electrodes^32^. To confirm that *S. oneidensis* could also transfer electrons to PEDOT:PSS via riboflavin-mediated mechanisms, we added riboflavin to abiotic bioreactors and the bioreactors containing the Δ*bfe* strain. While there was no current change in the abiotic reactors, the current sharply rose 36-fold and 87-fold in the bioreactors containing the Au and PEDOT:PSS/Au electrodes, respectively. Thus, we conclude that flavin-mediated transfer to PEDOT:PSS can also occur. The ability for *S. oneidensis* to transfer current to PEDOT:PSS films, with or without riboflavin shuttles, means that bacteria internal to the MCBF structure (Fig. 4d) are likely to produce current, thereby enhancing volumetric current production.

### PEDOT:PSS electrodeposition increases biotic current

**Figure 6:**
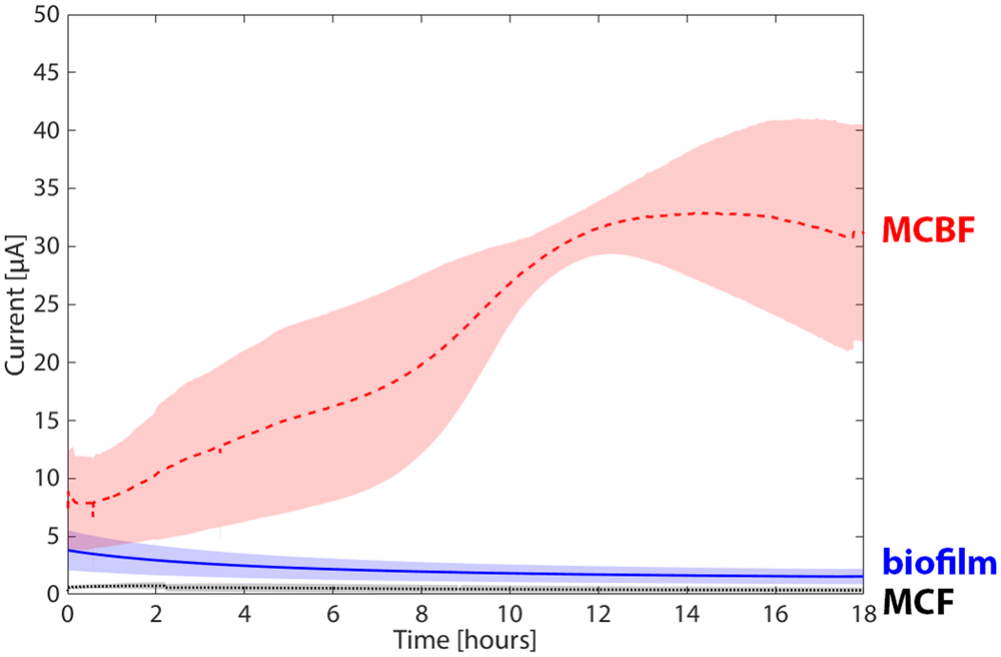
MCBF bioreactors produce greater biotic current than those using a native biofilm on unmodified CF. Chronoamperometric characterization of MESs based on MCBF (red dashed line) and UCF (blue solid line) using *S. oneidensis* metabolizing lactate, and abiotic MCF reactors (black dotted line). Light red, blue, and black colored bands indicate the standard deviation in current from three bioreactors, respectively.

To determine the improvement that our electrode modification process made on overall current output, we tested three samples of MCBF, MCF, and UCF anodes, the latter with native biofilm growth, in three-electrode MES configurations (MCBF-MES, MCF-MES, and UCF-MES, respectively). The electrodes under test were poised at constant 200 mV versus the Ag/AgCl reference electrode collecting the current originating from the bacterial electron transfer, while lactate served as the metabolic substrate. In the MCF-MES, where no bacteria were present, only a background current of 0.25 µA was observed, indicating the lactate and media did not transfer electrons to PEDOT:PSS. When a native biofilm was present, the UCF-MESs delivered a maximum current of 3.8 μA that dropped to a steady state current of 1.5 μA (Fig. 6). In contrast, MCBF-MESs produced significantly higher current compared to UCF-MESs, with a 20-fold increase in steady-state current. We conclude that this additional current is of bacterial origin, given the very low current production seen in the MCF-MESs. The current in the PEDOT-based bioanode gradually increased with time, peaking between 14 and 15 hours of operation. During this period, there was no significant change in the planktonic bacterial density as measured by OD_600_ which was under detectable levels after 16 hours in both the MCBF-MESs and UCF-MESs. Furthermore, *S. oneidensis*, a facultative anaerobe, does not grow aerobically in M9 with lactate at 30 °C with 250 rpm shaking (data not shown), suggesting that under these conditions *S. oneidensis* would not grow. Therefore, we attribute the current increase not to bacterial growth, but rather to adaptation of MCBF-MESs to anaerobic conditions at 30 °C from its preparation at 4 °C under aerobic conditions. Supporting the above claim, signals measured in native biofilm-based UCF-MESs exhibited no increase over time (Fig. 6)^12,17^. The slight current decrease observed over time might be explained by the gradual desorption of surface-bound electron-shuttling flavins or detachment of the cells themselves from the CF^34^. The MCBF-MESs produced a significantly higher current than UCF-MESs with the same macroscopic volumetric form factor. We suggest that this improvement was achieved due to the added high electroactive volume of the PEDOT:PSS conductive matrix and the incorporation of a dense biomass of viable bacteria embedded within this 3D matrix. The MCBF’s increased current within the same form factor makes it better suited for deployment in environmental biosensing systems than unmodified CF, since the same compact volume will produce a larger current signal.

## Discussion

We have demonstrated a new method for encapsulating electroactive bacteria into dense, multilayer conductive bacterial-composite films (MCBFs) that produce a 20x higher current-per-volume ratio than a native biofilm at steady state operation. We achieved this performance by embedding bacteria into high-density nutrient-permeable conductive multilayer composites that allow bacteria from inner layers to contribute to current generation in addition to those attached to the outer surface. Below, we discuss the mechanisms that likely underlie the observed current and outline how MCBFs open new opportunities in energy production and biosensing.

Several lines of evidence indicate that lactate diffusion through the PEDOT:PSS scaffold is central to the 20-fold current increase seen in MESs with MCBFs. PEDOT:PSS is known for its high molecular permeability, so small nutrients like lactate (90 Da) should be able to diffuse to the innermost bacteria in the MCBF^35^. Further supporting this is our direct observation that the large dyes DAPI (277 Da) and Cy5 (761 Da) readily diffused throughout the PEDOT:PSS structure for straining (Fig. 4). Also, the migration of other small molecules such as neurotransmitters similar in size to lactate have been reported to diffuse through PEDOT:PSS^36–39^. Lastly, if nutrient diffusion was blocked through PEDOT:PSS, we would expect only the outermost bacteria in the MCBF could contribute to current production. This scenario would cause the current levels in MCBFs and UCFs to be very similar, in contrast to the 20x difference in current that we observed. Taken together, these observations strongly suggest PEDOT:PSS permits lactate diffusion.

Data presented here also suggest that the bacteria embedded throughout the bacterial-composite film are contributing to current production. First, the bacteria within the composite film are viable, and thus capable of producing current. Second, our work shows that *S. oneidensis* can transfer electrons to PEDOT:PSS via both DET and riboflavin-mediated mechanisms. Thus, the bacteria internal to the MCBFs are surrounded by a conductive matrix that can directly transfer their electrons to the CF substrate or shorten flavin shuttling distances to further enhance electron transfer rate. In sum, our results suggest that these characteristics increase current production beyond superficial electron transfer to volumetric current production throughout the structure, accounting for the large increase in current production.

Previous work has used a variety of approaches to improve current density in MESs, which is surveyed in Table 1. Although far from comprehensive, this survey shows that despite the diversity of materials used to produce MESs, most approaches ultimately increase anode surface area (by using a large surface area material) and improve cell attachment. To improve cell attachment, the anode surface is usually either chemically modified or coated by a composite material or polymer. Although these strategies to improve cell attachment do increase current production, significant amounts of bacteria are not in direct contact with the electrode surface, resulting in sub-optimal electron transfer efficiency. Our bacterial-composite structure stands in contrast to these techniques by connecting many cells throughout a three-dimensional bacterial composite to the anode through a direct conductive path, which is a relatively new emerging strategy in the MES literature with consistently higher performance. Our current density increase of 20x is significantly larger than the improvement reported by most other works (Table 1). For the sake of comparison, we calculated the current density increase as either the ratio between steady state currents reported by the paper, or by comparing the largest currents reported by polarization curves. Importantly, our electropolymerization deposition strategy results in a relatively fast fabrication time, on the order of 16 hours, whereas the highest current density improvement reported by a bacterial composite requires 9 days of incubation for assembly of reduced graphene oxide particles^40^. Furthermore, our technique is unique among existing 3D bacterial composite approaches because it is the only one that uses PEDOT:PSS, an organic polymer with broad adoption in bioelectrochemical device fabrication. On the basis of these comparisons, we suggest that 3D bacterial composite approaches are worth additional investigation, especially with PEDOT:PSS as a main component.

**Table 1:** Comparison of MES anode modifications and current density improvements.

Strategy	Description	Anode substrate	Inoculant	Current density improvement	Ref.
3D bacterial composite	electropolymerized PEDOT:PSS-based bacterial-composite	carbon felt	*S. oneidensis* MR-1	20x	This work
	bacteria captured by biologicially reduced graphene oxide network	carbon cloth	*S. oneidensis* MR-1	25x	^40^
	iron(II,III) oxide/carbon nanotube nanocomposite co-cultured with bacteria, magnetically assembled	carbon paper	*E. coli* K12	18x	^44^
	bacteria individually coated with PPy	carbon cloth	*S. oneidensis* MR-1	12x	^17^
	bacteria embedded in graphite particle/PPy matrix	carbon cloth	*S. oneidensis* MR-1	7x	^45^
	PMBVF/PVA hydrogel surrounding bacteria	ITO	*S. oneidensis* MR-1	5x	^46^
Improve native biofilm conductivity	iron(III) oxide nanocolloid doped biofilm	ITO	*S. loihica* PV-4	80x	^47^
	poly(vinyl alcohol-*co*-polyethylene) nanofibers	PPy/PET	*E. coli* K12	9x	^16^
	gold nanoparticle doped biofilm	carbon plate	*G. sulfurreducens*	1.4x	^12^
Chemical treatment of anode	oxygen plasma	carbon paper	wastewater	4x	^48^
	ammonium nitrate	carbon mesh	wastewater	2x	^49^
	nitric and sulfuric acid	carbon cloth	wastewater	1.5x	^50^
	ammonia gas	carbon cloth	wastewater	1.2x	^51^
	ethylenediamine	activated carbon felt	pond sediment	1.2x	^52^
Composite material or polymer coating	ruthenium oxide nanoparticles	carbon felt	*S. decolorationis* S12	13x	^53^
	graphene/polyaniline foam	graphene foam	*S. oneidensis* MR-1	9x	^54^
	reduced graphene oxide/manganese oxide particles	carbon felt	MFC effluent	3x	^55^
	carbon nanotubes and chitosan	carbon paper	wastewater	1.7x	^56^
	electropolymerized PEDOT	carbon paper	*S. loihica* PV-4	1.6x	^57^
	electrospun PEDOT nanofibers	carbon cloth	*S. oneidensis* MR-1	N/A	^25^

This novel fabrication method will open new opportunities to further miniaturize portable MES platforms for environmental biosensing and create advanced bioelectrochemical sensors based on organic transistors. For example, a recently reported portable bioelectronic sensing system (BESSY) produces a baseline signal level of 6.31 μA cm^−3^, whereas our MCBF achieves 32.87 μA cm^−3^. Therefore, the MCBF would offer the same SNR if it were reduced to a volume of 0.38 cm^3^, a 5.2x reduction in anode volume from BESSY. Additionally, organic electrochemical transistors (OECTs) have been successfully used for local amplification of biological signals in organs, tissues and even individual cells, including in plants *in vivo*, and could be used to increase MES-based sensor sensitivity^21,22^. The active channel of most OECTs is based on PEDOT:PSS, therefore our technique is directly applicable to such device structures. Lastly, high throughput and large-scale printing of PEDOT:PSS patterns on various substrates allows substantial cost reduction in manufacturing of the sensors^41^. Most excitingly, our MCBFs move towards precise control of the structure of electroactive bacteria-based materials for novel biohybrid electronic devices.

## Conclusion

In this study we developed a functional multilayer bacteria-PEDOT:PSS biohybrid material for an anode that can electrically interface with bacteria regardless of their ability to form biofilms. This bacterial-composite anode produces 20-fold higher current density compared to standard porous carbon felt-based MESs employing *S. oneidensis* MR-1 in steady state operation. The presented method is scalable, preserves cell viability, can be expanded to other bacterial species, and opens the door to miniaturized, field-deployable MESs for biosensing and energy production. Furthermore, this work uses materials easily integrated with organic electrochemical transistors, making possible the direct integration of living material into miniaturized electronic devices for a new class of biohybrid electronics.

## Experimental Section

### Strains and growth conditions

All strains used in these experiments were derived from wild type *S. oneidensis* MR-1^42^. In addition to WT *S. oneidensis*, we used Δ*mtrB*, which lacks the transmembrane porin MtrB that facilitates the interaction between MtrA and MtrC and is therefore deficient in current production, and Δ*bfe* which lacks the bacterial flavin adenine dinucleotide (FAD) exporter, resulting in a severe decrease in extracellular riboflavin levels^32,43^. Cultures were inoculated from frozen glycerol stocks into 250 mL Erlenmeyer flasks containing 50 mL Luria-Bertani (LB) medium and grown overnight at 30 °C with 250 rpm shaking. After overnight growth, the cells were harvested by centrifugation at 5200 RCF at 4 °C for 10 minutes and washed twice with M9 medium. Finally, the cell pellet was resuspended in 1 mL M9 medium to the desired cell density. This bacterial suspension was kept on ice during anode preparation.

### Embedding *S. oneidensis* into electropolymerized PEDOT:PSS on CF anodes

Preparation of the electrodes was performed at 4 °C to maintain the viability of the bacteria throughout the electropolymerization process. To prepare the electropolymerization solution (EPS), poly(sodium 4-styrenesulfonate) (PSS) (Sigma Aldrich) and EDOT (3,4-ethylenedioxythiophene) (Sigma Aldrich) were dissolved together in 200 mL M9 medium, to a final EDOT concentration of 10 mM and EDOT/PSS weight ratio of 0.05. Then, electrodes were cut from 6.35 mm thick CF into 1.0 cm × 1.0 cm squares, and each one was soaked in 1 mL of M9. Each CF electrode was lightly compressed to remove trapped air bubbles. The electropolymerization platform consisted of a 6-well cell culture plate and custom 3D-printed stage for holding the electrodes, a peristaltic pump for the infusion of EPS, and a multi-syringe pump for infusion of bacterial culture (Fig. 1a). This setup allowed for the preparation of six anodes in parallel. Holes 2 mm in diameter were drilled through the center of each well to promote drainage. Pieces of 0.25 mm thick titanium foil (Sigma Aldrich), sheared into 25 mm by 150 mm strips, were wrapped into cylindrical shapes and inserted into each well to act as a counter electrode. To slow the diffusion of EPS and bacteria from the CF during electropolymerization, 20 mL of melted 1% agarose in M9 medium was added to each well. A soaked CF electrode square was then placed in the center of each well together and a titanium wire was inserted into the CF electrode before the agarose solidified. The Ti wire facilitated electrical connection to the potentiostat working electrode channel. After the agarose hardened around the CF electrode, a circular plug was removed from the agarose up to the electrode using a 4 mm biopsy punch connected to an aspirator. The plug removal left behind a small fluid reservoir above the CF enabling EPS to be directly introduced to the surface of the CF electrode (Fig. 1a). To promote drainage, a second hole was removed from the backside of the electrodes using a 2 mm biopsy punch connected to an aspirator. Finally, an Ag/AgCl reference electrode was held in contact with the agarose/M9 solution of each well, held into place by the custom stage (Fig. 1b). A peristaltic pump was used to continuously introduce fresh EPS during the overnight electropolymerization procedure. Each well had an independent line running to it from the central EPS reservoir. After priming the lines, the flow rate was set to 1.5 mL hour^−1^ to slowly introduce new EPS to the reservoir directly above each electrode. A separate line was used to introduce the bacterial suspension, which was prepared to an optical density at 600 nm (OD_600_) between 60–80, into a fluidic T-junction near the wells for mixing and to minimize time in contact with monomer EDOT. Multiple cultures were prepared the same way if more electrodes were to be prepared. A multi-syringe pump was used to deliver the bacteria to the fluidic T-junction, set to an infusion rate of 0.100 mL h^−1^. The large discrepancy in flow rates between the EPS and bacterial suspension ensured a high PEDOT-to-bacteria ratio as well as facilitated fluid mixing in the T-junction. Orthogonal views of the system are shown in Suplementary Figure S3. Once flow was established, the potentiostat was set to chronoamperometry mode, with the working electrode set to 1.0 V versus the Ag/AgCl reference. The current was recorded as the electropolymerization proceeded for 12–18 hours. When the electropolymerization was completed, the electrodes were disconnected, and the modified anode was cut out of the agarose with a scalpel. Excess agarose gel was removed from the sides of the anode, leaving behind an agarose shell approximately 2 mm thick to improve the mechanical stability of the electroplated CF electrode.

### Viability assay

Immediately after electropolymerization, live/dead analysis was performed on randomly selected samples. The anode was removed from the agarose well, and the excess agarose was removed by careful shaving with a scalpel. The anode was cut in two by a razor blade, and each half was gently washed with M9 medium to remove EPS and unbound bacteria. The samples were then immersed in 1.0 mL of M9 in a microcentrifuge tube. One of these tubes was submerged into a water bath at 70 **°**C for 15 minutes to heat-kill the bacteria, while the other tube remained at room temperature. Once the heat treatment was complete, the samples were moved to a glass-bottom 6-well plate (Mattek Corporation, P06g-1.5–20-F) for staining and subsequent imaging. A LIVE/DEAD^®^ BacLight^™^ Bacterial Viability Kit (Thermo Fisher Scientific, Product number L13152) was used to stain the bacterial-composite films attached to the anodes. SYTO 9 dye and propidium iodide were both added to M9 to a final concentration of 10 µM and 60 µM, respectively. One mL of the mixed dye solution was then pipetted onto each sample and incubated in the dark at room temperature for 15 minutes. Fluorescent images were acquired on a Zeiss LSM710 confocal microscope with an Axio Observer Z1. Two objectives were used: a 20x EC Plan-Neofluar objective (NA 0.3) with the pinhole set to 30 μm and a 40x EC Plan-Neofluar objective (NA 0.3) with the pinhole set 30 μm. SYTO 9 labeled all bacteria and was excited with a 488 nm Argon laser, and the emission was detected over the range 493–556 nm (*I*_SYTO9_). Propidium iodide labeled only those bacteria with damaged cell membranes, i.e. dead cells, and was excited using a 561 nm DPSS laser and its emission was collected between 593–719 nm (*I*_PI_). All samples were imaged with the same light intensity and detector gain. We calculated the dead:live ratio as *R* = *I*_PI_/*I*_SYTO9_. The heat-killed samples were used to set the laser intensity, exposure time, and pinhole size threshold for determining whether the bacteria were dead or alive in the test samples.

### Electrochemical characterization

Cyclic voltammetry and electrochemical impedance spectroscopy were performed using a BioLogic VMP-300 multipotentiostat. The anodes were scanned in the 6-well plates as described earlier. The anode was scanned from −0.5 V to 0.5 V with a scan rate of 40 mV sec^−1^. Four scans were measured, and the final scan was used to calculate the effective capacitance. Impedance spectra were collected with a stimulus of 10 mV peak-to-peak from 100 kHz to 32 mHz. Electrodes were characterized immediately before electropolymerization. After electropolymerization, the electrodes were gently washed with M9 and characterized again.

### Confocal laser scanning microscopy characterization

Slices of the modified anode were gently washed with M9 medium to remove EPS and unbound bacteria and then cut and immersed in 4% (v/v) formaldehyde solution in water. Samples were washed three times with MilliQ water and allowed to sit for 5 minutes in MilliQ water between each wash. Then, samples were placed in Tissue-Tek O.C.T. Compound (Sakura Finetek USA Inc.) and subsequently immersed in a dry ice ethanol bath for freezing. Samples were stored in a −80 **°**C freezer prior to imaging. When samples were ready to be imaged, they were removed from the freezer and allowed to warm while being sectioned into thin slices by a razor blade. These sections were stained in 14.3 μM DAPI (Thermo Fisher) in M9 medium and 1 μM Cy5 (Thermo Fisher) for 10 minutes. The DAPI stain labeled individual bacteria by their nucleus and the Cy5 provided a nonspecific agarose stain to enhance contrast with the carbon fibers. Each stained section was then placed on a glass coverslip and imaged either with a 20x EC Plan Apochromat objective (0.8 NA) or 63x Plan Apochromat oil immersion objective (1.4 NA). Confocal stacks of the sample were obtained using a 405 nm diode laser to excite DAPI and a 633 nm HeNe laser to excite Cy5, both using a 30 μm wide pinhole. The Cy5 fluorescence images were thresholded and then inverted to visualize the carbon fibers. The thickness of the MCBF structures were characterized on the confocal images as the distance from the carbon fibers to the point of farthest radial extent contained within the structures.

### Scanning electron microscopy characterization

Slices of the modified anode were gently washed with M9 medium to remove EPS and unbound bacteria and then cut and immersed in 4% (v/v) formaldehyde solution in water. Samples were washed three times with deionized water and allowed to sit for 5 minutes in deionized water between each wash. Samples were then set into a vacuum desiccator for 48 hours to dry before they were imaged in a field emission scanning electron microscope. The microscope used was the FESEM Ultra 55 set to an extra high-tension voltage level of 2 kV, under vacuum (5.0 × 10^−5^ mbar), with a working distance of 4–5 mm. Samples were held into place by conductive double-sided adhesive carbon tape (Electron Microscopy Sciences).

### Testing *S. oneidensis* ability to transfer electrons through PEDOT:PSS

Glass microscope slides were coated with 10 nm Ti, then 70 nm Au by standard electron beam physical vapor deposition (EBPVD) to be used as test anodes. A 2 cm × 4 cm section of these slides were coated by PEDOT:PSS by being immersed in electropolymerization solution and held at 1 V versus a Ag/AgCl reference electrode for 2 hours. After electropolymerization, the thickness of the PEDOT:PSS film was measured with a Dektak 3030 surface profiler. Additionally, the Au and PEDOT:PSS films were imaged with a Zeiss Gemini scanning electron microscope set to an extra high-tension voltage level of 1.5 kV, under vacuum (5.0 × 10^−5^ mbar), with a working distance of 2 mm. Then, each film was tested in a three-electrode single chamber bioelectrochemical reactor with a 10 mL solution volume (Supplementary Figure S11). Reactors were filled with M9 medium + 40 mM lactate and the film under test was set to 200 mV above the reference with a BioLogic VMP-300 multipotentiostat. After 30 minutes to allow the current to stabilize, the bacteria were injected to OD_600_ = 1.7. The experiment was performed with WT *S. oneidensis* MR-1, Δ*mtrB*, and Δ*bfe*. After 20 hours of incubation, 10 μM riboflavin was added to both the abiotic and Δ*bfe* reactors to determine the riboflavin-mediated electron transfer.

### Microbial fuel cell setup and biotic current measurement

To compare the performance of MCBFs to native biofilms, a biofilm of *S. oneidensis* was grown on the surface of an unmodified CF electrode. A three-electrode single-chamber bioelectrochemical reactor with a 250 mL solution volume was used. Reactors were filled with M9 and autoclaved at 121 °C for 20 min. A CF working electrode, 2.5 cm × 2.5 cm × 0.635 cm was inserted and connected to the potentiostat by a Ti wire threaded along its length. The counter electrode was a segment of Ti wire and the reference was an Ag/AgCl electrode. The potential was kept at 200 mV above the reference with a BioLogic VMP-300 multipotentiostat. The reactor was made anaerobic by continuous sparging of nitrogen gas. Once the reactor was prepared, a concentrated culture of *S. oneidensis*, grown overnight in 50 mL 2xYT medium (Sigma Aldrich) at 30 °C with 250 rpm shaking, was washed thrice in M9 and injected into the reactor to a final OD_600_ of 2.0. This culture was maintained for two days at room temperature to allow the bacteria to form a native biofilm on the surface of the unmodified CF. Then, the electrode was removed and gently dipped into M9 to wash unattached cells before starting chronoamperometry and cut to 1.0 cm × 1.0 cm dimensions to be comparable to the MCBF size. To compare the performance of the MCBF to a native biofilm, chronoamperometric measurements of the anodes were carried out in triplicate in three-electrode single-chamber bioelectrochemical reactors with 140 mL volumes. The working electrode used was either a modified MCBF-CF electrode, an abiotic MCF, or a native biofilm on unmodified CF as prepared by the method described in the previous paragraph. Bioreactors were continuously purged with nitrogen gas to establish anaerobic conditions, lactate was added to 40 mM, and the reactors were moved to 30 °C. Current was averaged and measured every 30 seconds. At the end of the experiment, the OD_600_ of a sample of supernatant was measured to ensure that no growth or major detachment of biomass occurred during chronoamperometry.

## Electronic supplementary material

Supplementary Information
