# Supplementary material for: PEDOT:PSS-based Multilayer Bacterial-Composite Films for Bioelectronics
Source: Sci Rep. 2018 Oct 16;8:15293. doi: 10.1038/s41598-018-33521-9 (PMC6191412; doi:10.1038/s41598-018-33521-9)
Supplement: Supplementary file 1 — Supplementary Information [file 41598_2018_33521_MOESM1_ESM.pdf]

PEDOT:PSS-based Multilayer Bacterial-Composite Films for Bioelectronics

Tom J. Zajdel[+], Moshe Baruch[+], Gábor Méhes[+], Eleni Stavrinidou, Magnus Berggren, Michel M. Maharbiz, and Daniel T. Simon*, Caroline M. Ajo-Franklin*

*corresponding authors: daniel.simon@liu.se; cajo-franklin@lbl.gov
[+]these authors contributed equally to this work

Supplementary Information

1. Supplementary Table and Figures
2. Estimation of double layer capacitance from cyclic voltammetry
3. Electrochemical impedance spectroscopy and analysis
4. References

Supplementary Table and Figures

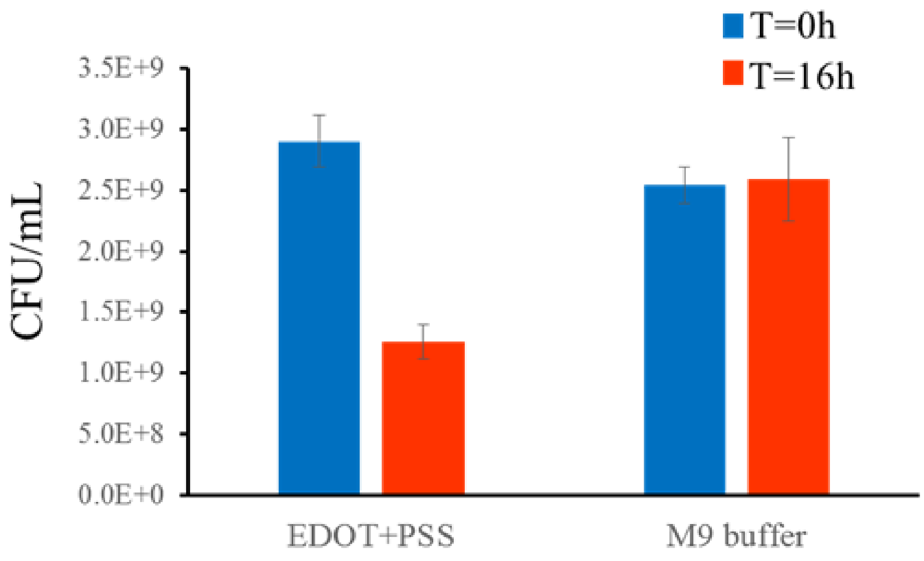

Figure S1. Prolonged exposure to 10 mM EDOT monomer reduces the viability of *S. oneidensis* MR-1. Colony forming units (CFUs) measured before and after 16 hours of exposure to EDOT/PSS precursor and M9 buffer at 4 °C.

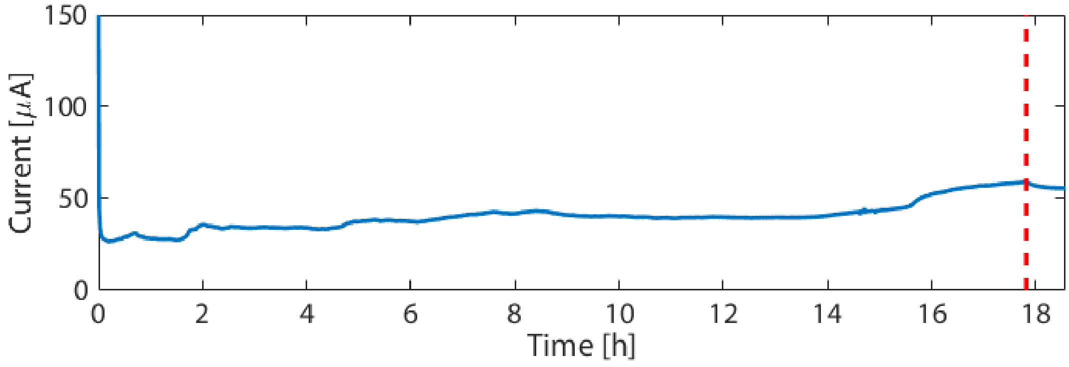

Figure S2. A representative electropolymerization current during potentiostatic PEDOT:PSS deposition during MCBF preparation. Total charge deposited was 1562 mC cm^{-3}. The dashed red line denotes when the flow of fresh electropolymerization solution was halted.

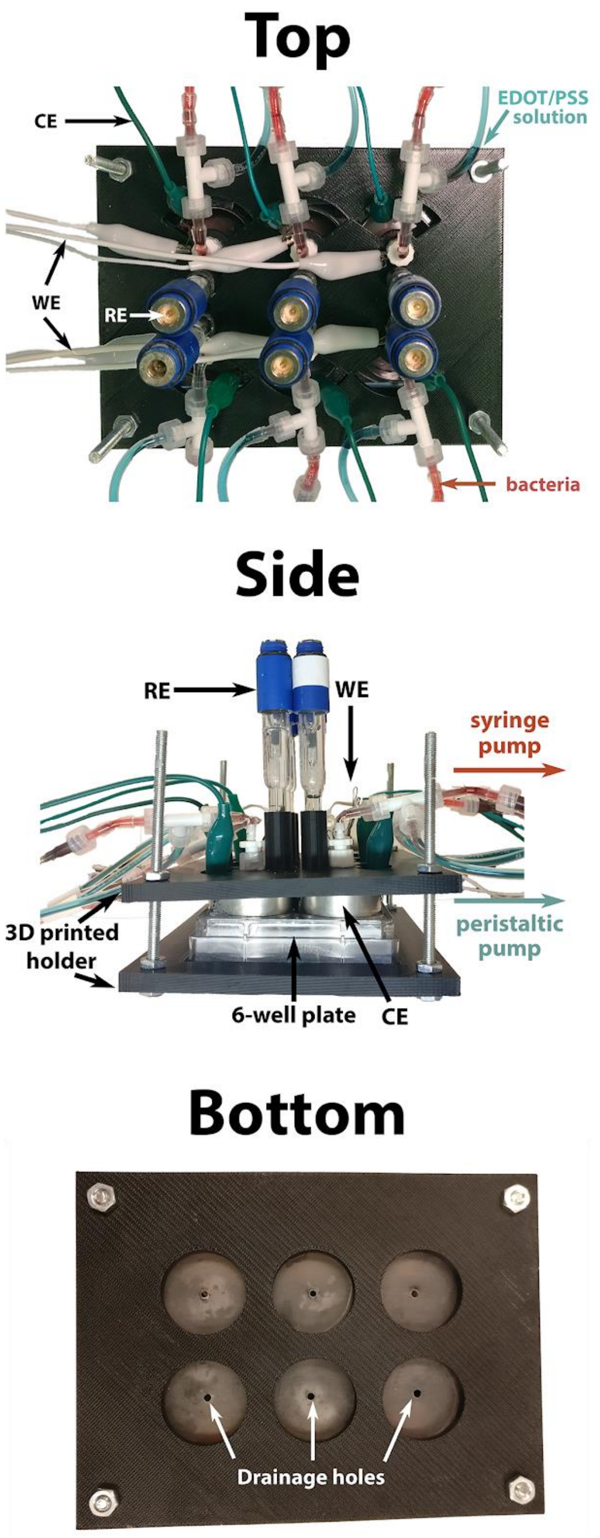

Figure S3. Orthogonal views of the MCBF preparation station.

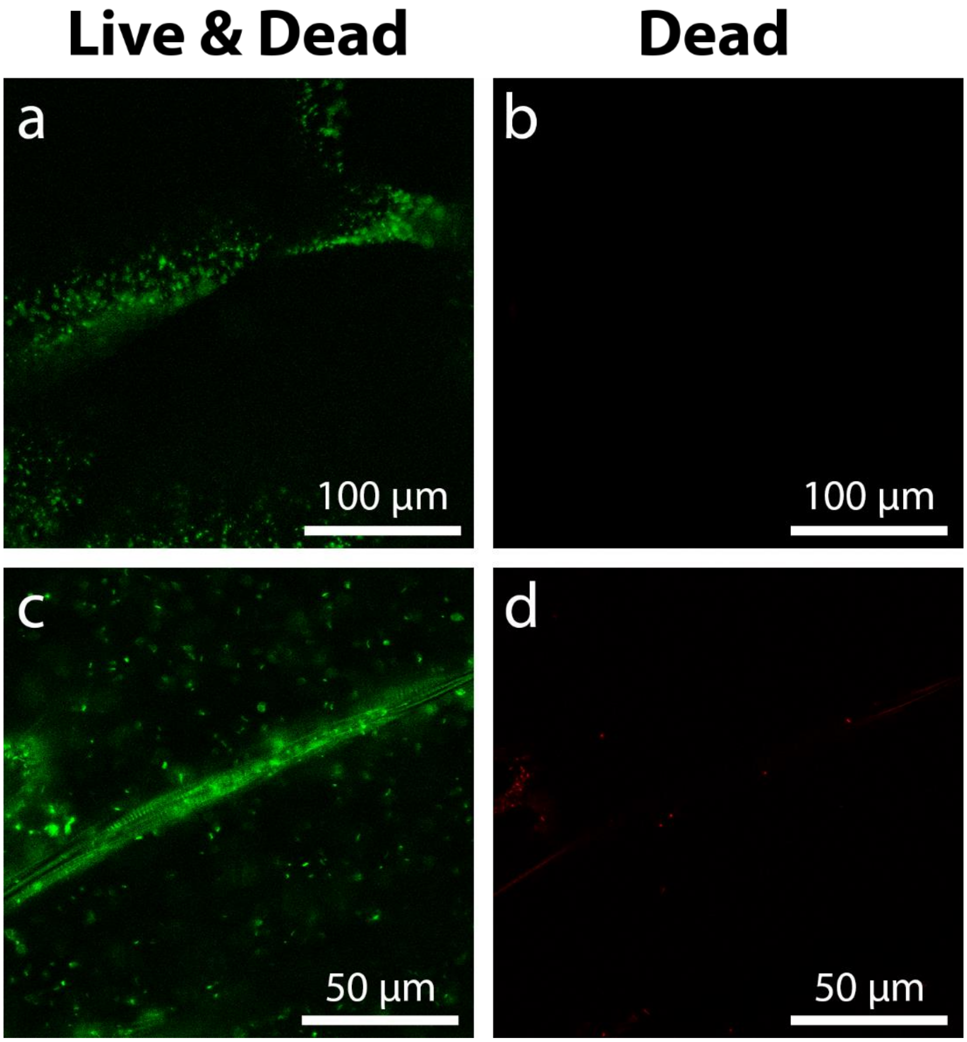

Figure S4. *S. oneidensis* MR-1 is viable after incorporation into MCBFs. Confocal microscopy images of a cross-sectioned MCBF stained by a live/dead assay at 20x magnification (a, b) and 40x magnification (c, d). Green fluorescence labels all bacteria (live and dead), red fluorescence labels only dead cells.

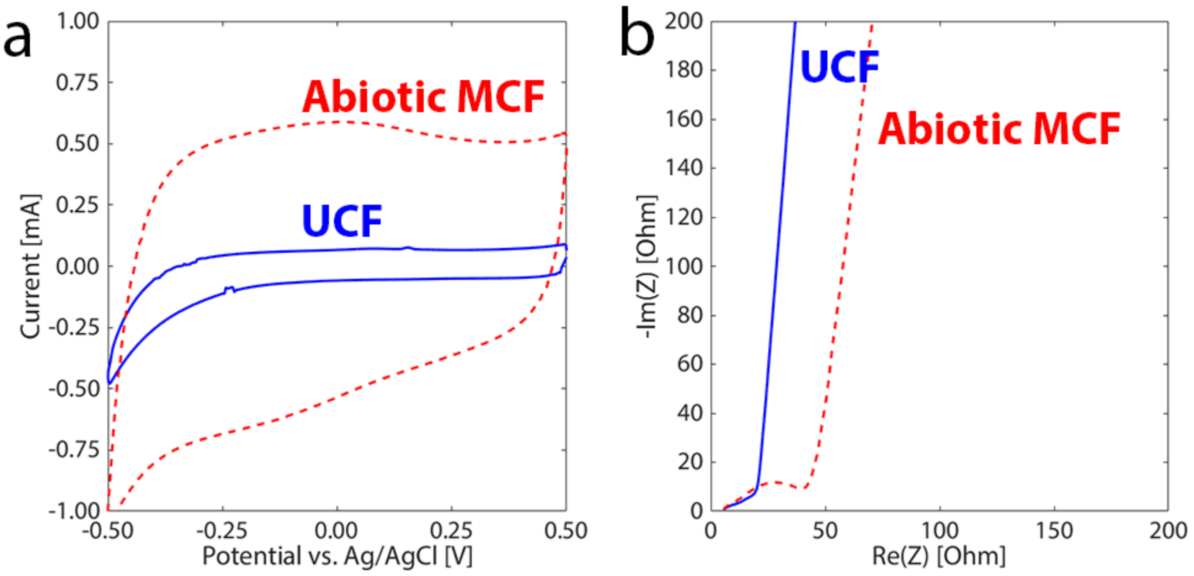

Figure S5. (a) Cyclic voltammograms and (b) Nyquist plots measured for unmodified CF before (blue), and for abiotic MCF after (dotted red) the electropolymerization process.

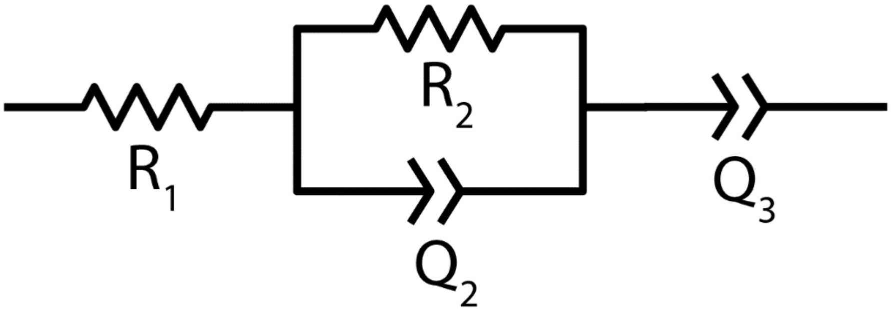

Figure S6. Equivalent circuit used to model the interfaces measured by EIS.

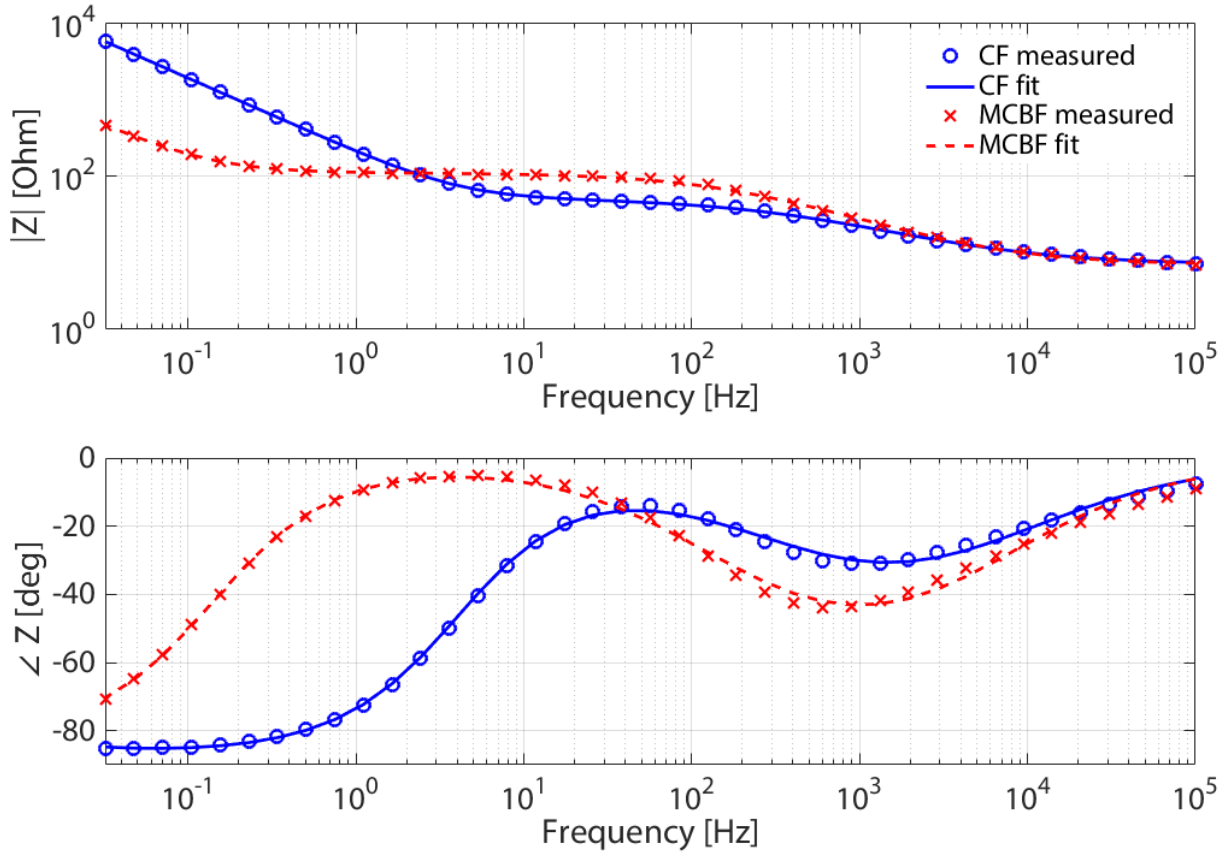

Figure S7. Bode plot of EIS spectra and fits before and after electropolymerization.

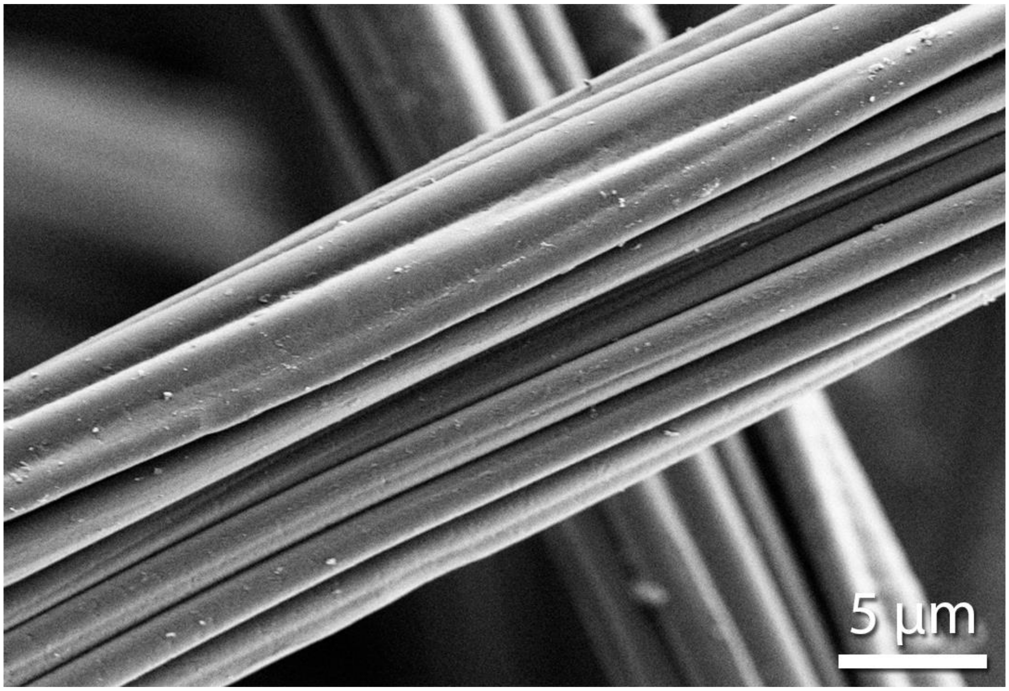

Figure S8. Scanning electron micrograph of modified carbon fiber (UCF).

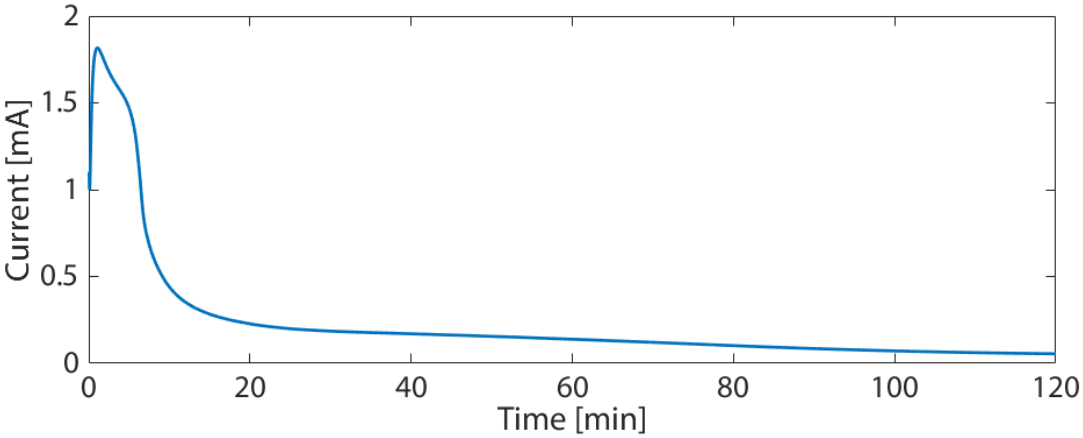

Figure S9. A representative electropolymerization current during potentiostatic PEDOT:PSS deposition on a gold thin film. Total charge deposited was 0.206 mC cm^{-2}.

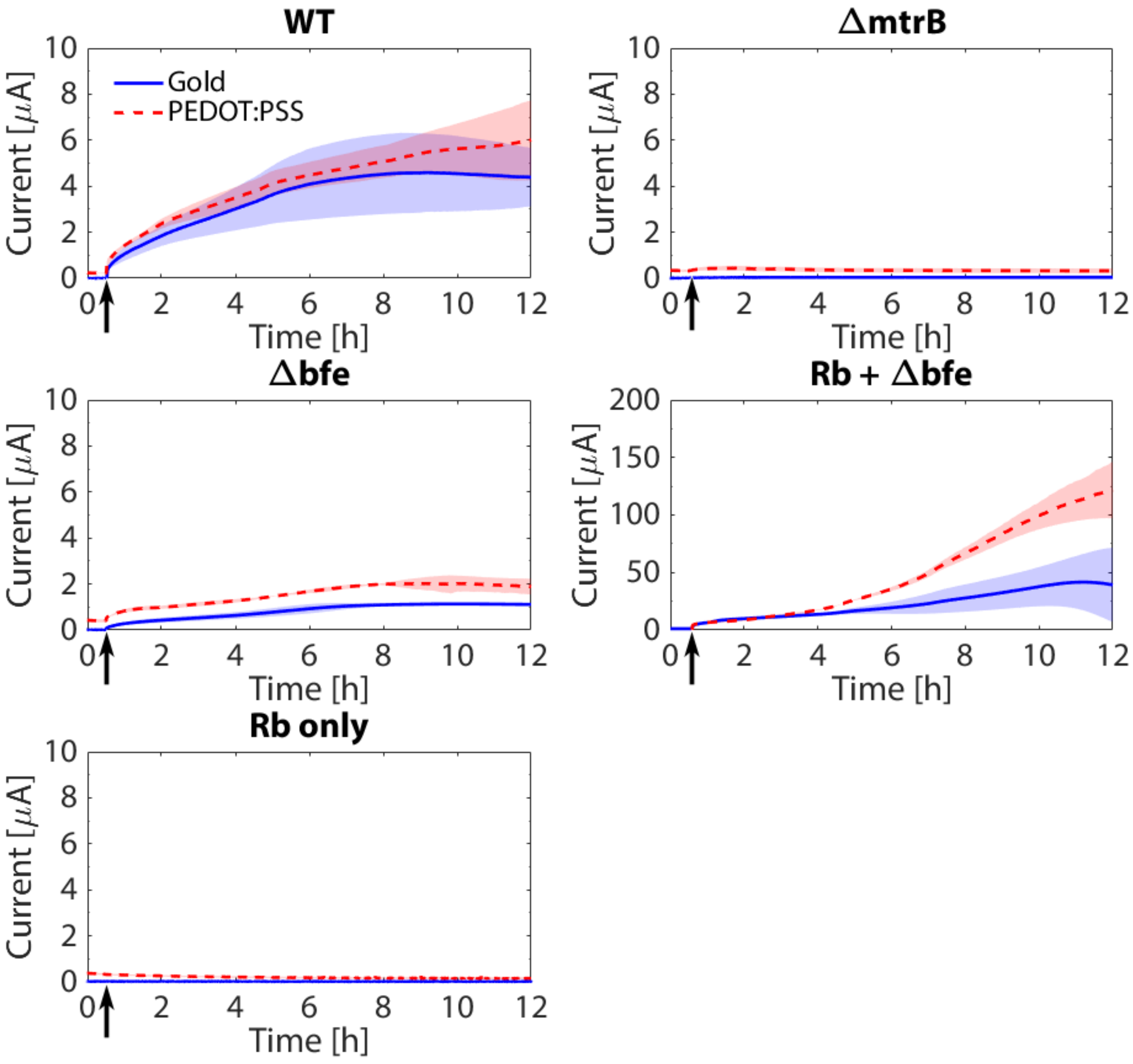

Figure S10. *S. oneidensis* transfers current to PEDOT:PSS directly and through riboflavin-mediated electron transfer. Chronoamperometric characterization of MESs based on MCBF (red) and UCF (blue) using *S. oneidensis* (WT, Δ*mtrB*, and Δ*bfe*) metabolizing lactate. Riboflavin (Rb) was added to the Δ*bfe* reactor and another reactor without bacteria. Light red and blue colored bands indicate the standard deviation in current from two bioreactors, respectively. Arrows denote the addition of bacteria (WT, Δ*mtrB*, and Δ*bfe*) or Rb (Rb + Δ*bfe* and Rb only).

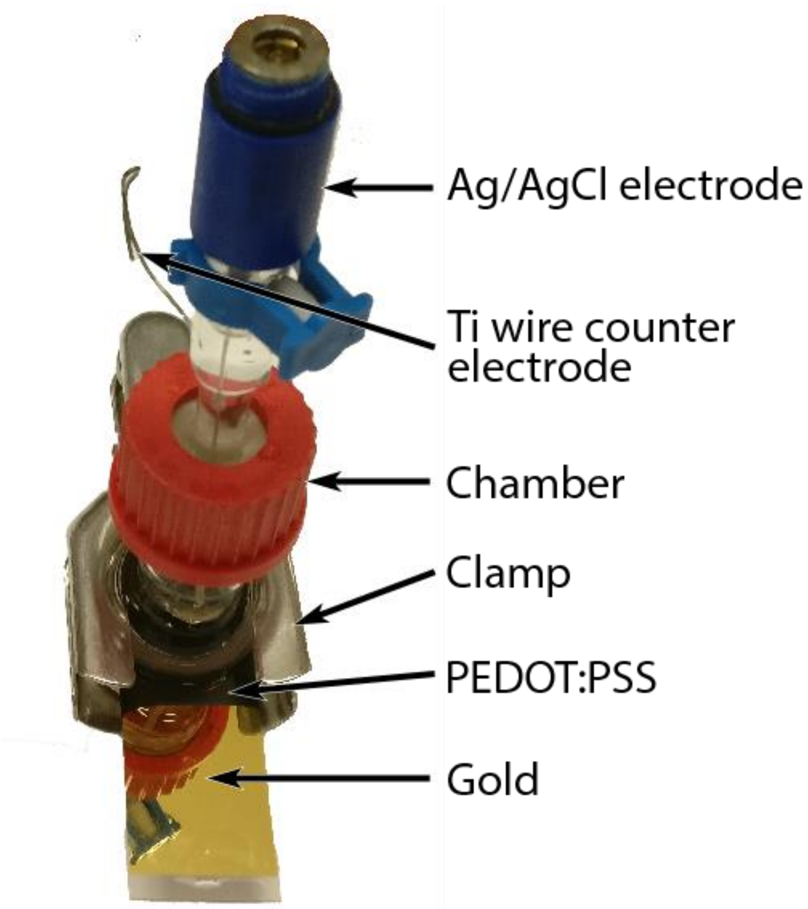

Figure S11. Reactor for measuring current transfer to gold and PEDOT:PSS thin films.

Table S1. Parameters from EIS spectra fit to equivalent circuit model.

Parameter	Units	Before Modification	After Modification
R_1	Ω	6.99	6.90
R_2 (R_{CT})	Ω	42.72	102.7
Q_2	$Fs^{\alpha-1}$	1.188×10^{-4}	5.855×10^{-5}
α_2	-	0.683	0.743
Q_3	$Fs^{\alpha-1}$	8.345×10^{-4}	1.040×10^{-2}
α_3	-	0.965	0.940

2. Calculation of double layer capacitance from cyclic voltammetry

To estimate the change in double layer capacitance and effective surface area resulting from the electropolymerization process, cyclic voltammetry (CV) was performed. The capacitance was estimated from the CV by the following formula adapted from other studies:[1—4]

$$C_v = \frac{\frac{1}{2}\oint i d\phi}{\nu \Delta\phi}\frac{1}{V} \tag{S1}$$

In Equation (S1), C_v is the volumetric capacitance [F cm^{-3}], i is the current [A], ν is the scan rate [V s^{-1}], ψ is the potential [V], and V is electrode volume [cm^3]. This equation estimates the capacitance by computing the average charge stored by the double layer capacitance throughout one full cycle of the CV, dividing by the potential window ($\Delta\psi$), and normalizing the result to the electrode volume. The electrode volume used in this study was 0.635 cm^3.

3. Electrochemical impedance spectroscopy analysis

To determine the effective resistance change of the anode after electropolymerization, EIS spectra were measured from 100 kHz to 32 mHz with a stimulus amplitude of 10 mV. Four data points were averaged for each frequency collected. Figure S7 shows the equivalent circuit used to fit the spectra both before and after electropolymerization.

The element R_1 models the solution resistance, R_2 and Q_2 model the anode-solution interface, and Q_3 models the low frequency behavior of the interface. Q_2 and Q_3 are modeled as constant phase elements (CPEs) with impedance given by:

$$Z_Q = \frac{1}{Q(j\omega)^{\alpha}} \tag{S2}$$

In equation (S2), ω is the stimulus angular frequency [radians s^{-1}], j is the imaginary number, and Q [$Fs^{\alpha-1}$] and α are parameters describing the CPE. The equivalent circuit model was fit to the EIS spectra using the ZFit functionality of BioLogic's EC-Lab Express software. The built-in randomized simplex optimization was used with 20000 iterations and randomization. The resulting parameter values fit to a representative sample are shown in Table S1. In Figure S8, a Bode plot of the experimental impedances are plotted alongside these fits. From this plot, a reasonable fit is observed. The chi-square values for the fits are 0.0227 and 0.0604 for before and after electropolymerization, respectively.

4. References

1. Byon, H. R., Lee, S. W., Chen, S., Hammond, P. T. & Shao-Horn, Y. Thin films of carbon nanotubes and chemically reduced graphenes for electrochemical micro-capacitors. *Carbon N. Y.* **49,** 457–467 (2011).
2. Pell, W. G., Conway, B. E. & Marincic, N. Analysis of non-uniform charge/discharge and rate effects in porous carbon capacitors containing sub-optimal electrolyte concentrations. *J. Electroanal. Chem.* **491,** 9–21 (2000).
3. Wang, H. & Pilon, L. Physical interpretation of cyclic voltammetry for measuring electric double layer capacitances. *Electrochim. Acta* **64,** 130–139 (2012).
4. Yan, J. *et al.* Electrochemical properties of graphene nanosheet/carbon black composites as electrodes for supercapacitors. *Carbon N. Y.* **48,** 1731–1737 (2010).
